# Lecithin derived from ω-3 PUFA fortified eggs decreases blood pressure in spontaneously hypertensive rats

**DOI:** 10.1038/s41598-017-12019-w

**Published:** 2017-09-28

**Authors:** Dorian Nowacki, Helena Martynowicz, Anna Skoczyńska, Anna Wojakowska, Barbara Turczyn, Łukasz Bobak, Tadeusz Trziszka, Andrzej Szuba

**Affiliations:** 1 Department and Clinic of Internal and Occupational Diseases and Hypertension, Wroclaw Medical University, Borowska 213, Wroclaw, 50-556 Poland; 2 Department of Animal Products Technology and Quality Management, Wroclaw University of Environmental and Life Sciences, J. Chełmońskiego 37, Wroclaw, 51-630 Poland; 3 Division of Angiology, Faculty of Health Science, Wroclaw Medical University, 5 Bartla St., Wroclaw, 51-618 Poland; 4 Department of Internal Medicine, 4th Military Teaching Hospital, Wroclaw, Poland

## Abstract

Hypertension is the most common risk factor for stroke, coronary heart disease and heart failure, which are the leading causes of death worldwide. Dietary patterns and supplements intakes are becoming important factors in the hypertension. The aim of this study was to estimate the effects of new generation egg yolk phospholipids rich in lecithin (SL) esterified with omega-3 and omega-6 fatty acids on blood pressure in hypertensive rats (SHR). Here we have reported that lecithin (SL) derived from egg yolk lowers blood pressure in pathology of hypertension. The SHR rats treated with SL had significantly lower blood pressure than control group (157/104 vs. 178/121 mmHg; P < 0.05) and down-regulated mesenteric artery over-response to norepinephrine and potassium chloride, giving similar arterial response as for normotensive Wistar Kyoto rats (WKY). Hypertensive rats treated by SL demonstrated significantly lower serum level of inflammatory factors. This work also indicates that SL treatment lowers heart rate and reduces the serum level of oxidative stress marker - nitrotyrosine - by 30–34% in both hypertensive and normotensive animals. Phospholipids with lecithin derived from PUFA fortified eggs may be a valuable dietary supplement in prophylaxis of hypertension and in patients with hypertension, however, this requires further studies on humans.

## Introduction

Epidemiological studies show arterial hypertension as the leading risk factor of cardiovascular diseases. It is, additionally, one of the most frequently ailments in populations of developed countries^1^. Prognoses shows that in 2030 as much as 35.7% of the USA population will suffer from hypertension. High values of arterial blood pressure and related complications pose a serious medical challenge and a global issue from both economic and social perspective that needs to be faced by the contemporary medicine. Functional food and nutritional therapeutic interventions are increasingly often perceived as tools in the promotion and maintenance of health and in the prophylaxis of the diseases. An appropriate diet plays a significant role in the pathogenesis of arterial hypertension. The research with hypertensive patients demonstrates that DASH diet (Dietary Approach to Stop Hypertension), rich in fish, poultry, nuts, vegetables, fruit, low-fat dairy products and whole-grain products, affects a reduction in the mean values of diastolic and systolic blood pressure by 5.5 mmHg and 11.5 mmHg. In turn, in patients with high-normal blood pressure (ESH/ESC classification), the DASH diet decreased the values of diastolic and systolic blood pressure by 3.0 and 5.5 mmHg, respectively^2^. The enrichment of a diet of non-treated hypertensive patients with fish-derived ω-3 PUFAs in a dose of 15 g/day reduces values of diastolic and systolic blood pressure by 5.6 mmHg and 8.1 mmHg, respectively^3^. Fish are a common source of polyunsaturated fatty acids (PUFA), including docosahexaenoic acid (DHA) and (eicosapentaenoic acid) EPA. A well-balanced diet providing the body with sufficient quantities of PUFAs shall contain products of fish origin. The American Heart Association recommends PUFAs intake at 1 g/day (recommendation since 2010) to patients suffering from cardiovascular diseases. However, a high intake of sea fish poses the risk of the intake of high quantities of heavy metals like mercury. The severity of this problem is indicated by the Food and Drug Administration (FDA) Department that recommends exclusion of selected fish species from diets of pregnant or breastfeeding women^4^. Hence, a need for an alternative emerges of a PUFAs sources in diet. A solution may be found in hen eggs. They are very important natural food products considering their contents of polyunsaturated fatty acids, phosphatidylcholine, exogenous amino acids, lutein, xanthophyll, folic acid as well as B1 and B2 vitamins. Some studies suggest that frequent consumption of eggs may be a risk factor of diabetes and that it is correlated with increased mortality rate of diabetic patients^5,6^. These observations are not confirmed by some other large population surveys^7,8^. In addition, consumption of eggs may increase the risk of complications in diseases with pathology involving deterioration of the lipid profile^9^. It needs to be noticed that this opinion - though common for years - has not been corroborated in recent investigations. A met-analysis published in 2013 did not confirm any relationship between egg consumption and risk of cardiovascular diseases, however it suggested its effect on the increased risk of type 2 diabetes development^10^. Another study, demonstrated that the consumption of more than one egg a day was linked with lesser intensity of coronary artery atherosclerosis in coronography^11^. The effect of egg consumption on the cardiovascular risk has been highly controversial. Noteworthy is that the above-cited findings refer to standard eggs available in countries of Western civilization (i.e. characterized by high contents of saturated fatty acids and cholesterol) and to egg consumption in the aspect of an unhealthy diet, i.e. together with fried bacon.

In our research eggs were enriched in PUFAs via modifications of diets for laying hens. These involve natural methods of eggs enrichment that do not engage products of chemical synthesis nor genetic engineering techniques. Hens’ feed is enriched in natural products like algae, fish oil or linseed oil. Laying hens serve the function of a specific bioreactor that produces eggs with a desirable composition. Eggs of this type may be then used as functional foods, whereas biologically-active substances isolated from such eggs may be applied in the production of dietary supplements, nutraceuticals and drugs. Reduction of undesirable components and egg enrichment in omega-3 fatty acids not only diminishes the potential risk related to egg consumption but also may induce a beneficial effect on the lipid metabolism and course of many diseases. High-quality phospholipid fraction rich in lecithin (egg-lecithin; SL), omega-6 and omega-3 fatty acids (including DHA) was isolated from specially designed eggs (so-called new generation eggs). The aim of this study was to evaluate the effect of diet supplementation with an egg lecithin derived from new-generation of eggs on the development of arterial hypertension in spontaneously hypertensive rats (SHR).

## Results

### Blood pressure and heart rate

The SHR treated by SL (SHR-SL) had a significantly lower blood pressure than not treated SHR animals from the control group (157/104 vs. 178/121 mmHg; P < 0.05). Between the groups of normotensive rats (WKY-SL and WKY-C) there was no differences in mean levels of blood pressure as well as systolic and diastolic blood pressure (113/79 vs. 115/80 mmHg). Rats treated with egg-lecithin (SL) express a statistically significantly lower heart rate. SL affected a decrease in heart rate by 3.83% in both hypertensive (SHR) and normotensive (WHY) rats. All rats exhibited the same activity during measurements, what excludes a potential impact of activity on blood pressure and heart rate (Table 1).

**Table 1:** The mean levels of all parameters measured by telemetry for SHR treated with egg-lecithin (SHR-SL; n = 15), SHR not treated control group (SHR-C; n = 15), WKY treated with egg-lecithin (WKY-SL; n = 11) and WKY not treated control group (WKY-C; n = 11).

	SHR-SL Mean ± SEM	SHR-C Mean ± SEM	WKY-SL Mean ± SEM	WKY-C Mean ± SEM
Activity [counts/min]	2.20 ± 0.20	2.26 ± 0.19	2.03 ± 0.15	1.89 ± 0.17
Heart rate [BPM]	285.06* ± 5.21	296.41 ± 3.03	298.74* ± 2.96	310.63 ± 4.92
Pulse pressure [mmHg]	52.72 ± 1.52	56.31 ± 0.83	35.03 ± 0.44	33.56 ± 0.65
Mean pressure [mmHg]	129.98* ± 2.23	149.36 ± 0.91	96.02 ± 0.90	94.08 ± 1.00
Systolic blood pressure [mmHg]	156.65* ± 2.84	177.60 ± 0.99	115.25 ± 1.01	112.69 ± 1.06
Diastolic blood pressure [mmHg]	103.94* ± 1.56	121.29 ± 0.99	80.22 ± 0.77	79.12 ± 1.09

*P < 0.05, compared to the control group (SL vs. C).

### Basal perfusion pressure of the mesenteric artery

#### Difference between the groups

Significantly lower values of the basal perfusion pressure (PP) were observed at all stages of the perfusion procedure in the experimental group of SHR rats (SHR-SL), in comparison to the control SHR group (SHR-C). In the group of WKY rats receiving the phospholipid fraction (WKY-SL), significantly higher PP_1_ was observed only at the first stage of perfusion *ex vivo* of mesenteric artery in comparison with the control WKY group (WKY-C). At the successive stages of the perfusion, no significant differences were observed in the basal perfusion pressure (PP) between groups of WKY rats. No significant differences were shown between groups of WKY rats in the total PP levels taken from the whole perfusion analysis (PP_T_). In turn, in the SHR, the phospholipid fraction (SL) was found to significantly decrease the PP_T_ value (Table 2).

**Table 2:** Mean levels of basal perfusion pressure of the both rats’ strain (SHR and WKY) treated with egg-lecithin (SL) and not treated control group (C).

	**SHR-SL** (n = 15)	**SHR-C** (n = 15)	**WKY-SL** (n = 11)	**WKY-C** (n = 11)
	Mean ± SEM	Mean ± SEM	Mean ± SEM	Mean ± SEM
***PP***_***1***_ ***[mmHg]***	52.48* ± 1.09	62.04 ± 1.74	50.01* ± 1.56	41.30 ± 1.55
***PP***_***2***_ ***[mmHg] -*** * Infusion NE*	86.81*^A^ ± 2.72	101.32^A^ ± 2.67	58.95^A^ ± 1.94	60.01^A^ ± 2.68
***PP***_***3***_ ***[mmHg]-*** * Infusion NE* + *L-NOARG*	91.94* ± 3.07	114.16^B^ ± 3.70	54.22 ± 1.55	60.90 ± 2.78
***PP***_***T***_ ***[mmHg]***	76.75* ± 1.68	92.20 ± 2.04	53.52 ± 0.97	53.54 ± 1.47

PP_1_ [mmHg] – basal perfusion pressure in the first stage of the study. PP_2_ [mmHg] - perfusion pressure during the infusion NE, second stage of study. PP_3_ [mmHg] - basal perfusion pressure during the infusion NE + L-NOARG, third stage of study. PPT [mmHg] - mean basal perfusion pressure of all perfusions’ stages. *P < 0.05, compared to the control group (SL vs. C). ^A^P < 0.05, significant increase of perfusion pressure inside the group caused by NE infusion (PP_1_ vs. PP_2_). ^B^P < 0.05, significant increase of perfusion pressure inside the group caused by NE + L-NOARG infusion (PP_2_ vs. PP_3_).

#### Difference inside the group

In all investigated groups, result demonstrate a significant increase of PP values caused by NE infusion (Stage 1 vs. Stage 2: PP_1_ vs. PP_2_). After NE + L-NOARG infusion, a significant increase of PP was observed only in the SHR-C group, (Stage 2 vs. Stage 3: PP_2_ vs. PP_3_). In the remaining groups, NE + L-NOARG infusion had no significant effect upon PP levels (Table 2).

In addition, the correlation coefficient (*r* = *0*.*45*, *P* < *0*.*05*) in SHR shows a linear correlation between arterial blood pressure measured with the telemetric method and PP_T_ value, i.e. the basal perfusion pressure was increasing along with an increasing arterial blood pressure.

### Reactivity of mesenteric artery

No differences were found between WKY rats’ groups in mesenteric artery response to a series of norepinephrine injections (NE: 0.01–5.00 μg). Different response of the mesenteric artery was observed in the SHR strain. Significant differences were noticed in the SHR group receiving the phospholipid fraction (SHR-SL), in which the response to all NE doses (0.01–5.00 μg) was significantly lower than in the control group of SHR rats (SHR-C). Also at the last stage of the perfusion analysis, a significantly lesser KCl-induced increase of perfusion pressure was observed in SHR experimental group (SHR-SL vs. SHR-C; Table 3)

**Table 3:** Changes of perfusion pressure (ΔPP [mmHg]) in the mesenteric artery induced by norepinephrine and potassium chloride injections.

The pharmaceutical in injection (dose):	SHR-SL Mean ± SEM	SHR-C Mean ± SEM	WKY-SL Mean ± SEM	WKY-C Mean ± SEM
Norepinephrine (0.01 μg)	8.63* ± 1.58	26.47 ± 9.15	15.00 ± 3.73	21.18 ± 4.81
Norepinephrine (0.10 μg)	47.33* ± 7.70	77.80 ± 6.06	35.14 ± 5.71	38.77 ± 6.65
Norepinephrine (0.50 μg)	85.80* ± 12.07	151.60 ± 8.78	70.27 ± 9.03	66.50 ± 9.51
Norepinephrine (1.00 μg)	96.43* ± 14.21	189.97 ± 8.91	88.23 ± 9.95	80.00 ± 11.29
Norepinephrine (3.00 μg)	109.40* ± 16.54	203.83 ± 9.17	98.05 ± 13.72	91.14 ± 12.44
Norepinephrine (5.00 μg)	118.33* ± 17.62	204.47 ± 7 0.41	102.73 ± 13.87	84.95 ± 11.56
KCl 10.5 mg/ml	43.03* ± 4.36	56.93 ± 3.85	26.45 ± 3.45	35.36 ± 4.58

ΔPP for SHR treated with egg-lecithin (SHR-SL; n = 15), SHR not treated control group (SHR-C; n = 15), WKY treated with egg-lecithin (WKY-SL; n = 11) and WKY not treated control group (WKY-C; n = 11). *P < 0.05, compared to the control group (SL vs. C).

### Nitrotyrosine concentration in blood serum

**Figure 1:**
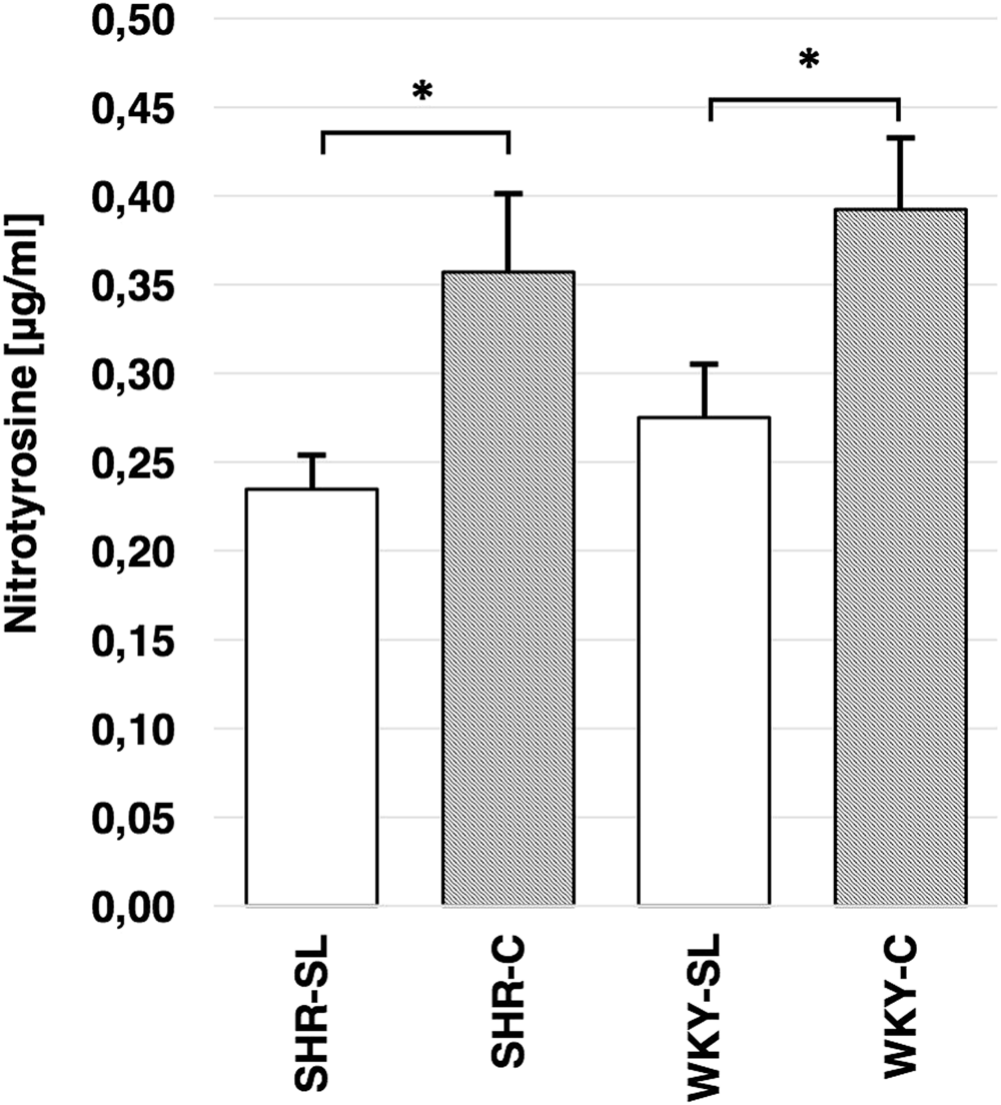
Mean concentration of nitrotyrosine [mg/ml] in blood serum of SHR treated with egg-lecithin (SHR-SL; n = 15), SHR not treated control group (SHR-C; n = 15), WKY treated with egg-lecithin (WKY-SL; n = 11) and WKY not treated control group (WKY-C; n = 11). *P < 0.005.

The phospholipid fraction (SL) significantly reduced the concentration of nitrotyrosine in blood serum of both rats’ strains: SHR and WKY. In the SHR group treated with egg-lecithin (SHR-SL), the analyzed marker of oxidative stress was lower by 34% than in the SHR-C group (0.23 ± 0.02 vs. 0.36 ± 0.04 μg/ml; n = 15; Fig. 1). In the WKY-SL group, the concentration of NT was lower by 30% compared to the WKY-C group (0.28 ± 0.03 vs. 0.39 ± 0.04 μg/ml; n = 11; Fig. 1).

### Cytokines and adhesive molecules level in blood serum

The SHR treated with egg-lecithin (SHR-SL) demonstrated lower concentrations of TNF-α, MCP-1, sICAM-1 and sE-selectin in comparison to the control SHR animals. In groups of normotensive rats (WKY), the egg-lecithin treatment had no significant effect upon levels of cytokines nor adhesive molecules (Table 4).

**Table 4:** Mean concentrations of cytokines and adhesive molecules in blood serum in SHR treated with egg-lecithin (SHR-SL; n = 15), SHR not treated control group (SHR-C; n = 15), WKY treated with egg-lecithin (WKY-SL; n = 11) and WKY not treated control group (WKY-C; n = 11). *P < 0.05, compared to the control group (SL vs. C).

	SHR-SL Mean ± SEM	SHR-C Mean ± SEM	WKY-SL Mean ± SEM	WKY-C Mean ± SEM
TNF-α [pg/ml]	5.40* ± 0.25	6.56 ± 0.28	6.91 ± 0.37	6.84 ± 1.21
MCP-1 [pg/ml]	1,491.07* ± 44.44	1,760.93 ± 56.46	1,235.91 ± 115.67	1,370.36 ± 160.15
sICAM-1 [ng/ml]	9.88* ± 0.68	12.61 ± 0.87	6.21 ± 1.16	6.66 ± 0.84
sE-selectin [ng/ml]	95.33* ± 1.57	124.87 ± 5.03	129.63 ± 6.74	135.33 ± 4.23

The correlations observed in hypertensive rats (SHR) indicate dependences between arterial blood pressure and MCP-1 concentration (*Spearman R coeff*. = *0*.*55*, *P* < *0*.*05)* and between basal perfusion pressure (PP) and MCP-1 concentration (*r* = *0*.*42*, *P* < *0*.*05*). A correlation was also observed in the SHR rats between concentrations of TNF-α, MCP-1, sE-selectin and the response of the mesenteric artery to four of the six doses of exogenous norepinephrine (MCP-1 and 0.01 μg NE, Spearman R coeff = 0.42, P < 0.05; MCP-1 and 0.1 μg NE, r = 0.38, P < 0.05; MCP-1 and 0.5 μg NE, r = 0.46, P < 0.05; MCP-1 and 1.0 μg NE, r = 0.48, P < 0.05; TNF-α and 0.5 μg NE, r = 0.51, P < 0.05; TNF-α and 1.0 μg NE, r = 0.50, P < 0.05; TNF-α and 3.0 μg NE, Spearman R coeff = 0.44, P < 0.05; TNF-α and 5.0 μg NE, Spearman R coeff = 0.41, P < 0.05; sE-selectin and 0.5 μg NE, Spearman R coeff. = 0.56, P < 0.05; sE-selectin and 1.0 μg NE, Spearman R coeff. = 0.57, P < 0.05; sE-selectin and 3.0 μg NE, Spearman R coeff. = 0.51, P < 0.05; and sE-selectin and 5.0 μg NE, Spearman R coeff. = 0.48, P < 0.05). In addition, our study demonstrated a positive correlation between heart rate and serum levels of TNF-α (*Spearman R coeff*. = *0*.*40*, *P* < *0*.*05)*, MCP-1 (*Spearman R coeff*. = *0*.*48*, *P* < *0*.*05*) and E-selectin (*Spearman R coeff*. = *0*.*51*, *P* < *0*.*05*).

## Discussion

To the best of our knowledge, there is no research on the effects of phosphatidylcholine (lecithin) on hypertension. Results reported in this work show the hypotensive effect of the egg phospholipids from new-generation eggs with lecithin as major component. Egg-lecithin (SL) was demonstrated to decrease mean values of blood pressure by over 19 mmHg in hypertensive rats (SHR). A lack of such effect on the normotensive rats’ strain (WKY) show that the beneficial effect of the egg-lecithin is manifested in the regulation of processes related to pathology of hypertension. Moreover, the correlation shows that decrease in the arterial blood pressure observed in SHR rats treated by the egg-lecithin (SHR-SL) was accompanied by a lower perfusion pressure of the mesenteric artery. The tension of the mesenteric artery of SHR-SL was statistically lower at all stages of the perfusion analysis (SHR-C vs. SHR-SL).

The positive impact of the phospholipid complex on blood pressure values in SHR rats may be determined by the presence of phosphatidylcholine as well as omega-3 and omega-6 fatty acids in the egg-lecithin (SL). Another importance is the appropriate ratio of omega-6 to omega-3 (ω-6/ω-3) fatty acids because the lower the ratio, the greater is its significance in the prevention of cardiovascular diseases. In a standard Western diet (Western Pattern diet) ω-6/ω-3 ratio shall range from 15/1 to 17/1. Ratio decreased to 4/1 reduce the mortality rate due to cardiovascular diseases by 70%^12^. In the analyzed egg-lecithin (SL), the ratio of omega-6 to omega-3 fatty acids was very low and reached 1.46.

A significant increase in perfusion pressure caused by the infusion of nitric oxide synthase inhibitor (N-ω-nitro-L-arginine; L-NOARG; Stage 2 vs. Stage 3: PP_2_ vs. PP_3_; Table 2) was observed in the not treated group of hypertensive rats (SHR-C). In turn, no significant response to L-NOARG infusion was observed in the group of hypertensive rats treated by egg-lecithin and in both normotensive WKY groups. This indicates that the function of endothelial nitric oxide synthase (eNOS) seems to be less significant in the regulation of endothelial processes in normotensive animals. Furthermore, it points to enhanced activity and greater role of eNOS in the regulation of arterial blood vessel tension in hypertension pathogenesis in SHR rats. The enhanced production of hypotensive nitric oxide (NO) in SHR rats may be deemed a natural protective mechanism induced in response to pressor processes in this experimental model^13^. Similar observations were made in Sabra hypertension-prone (SHP) rats, where the application of eNOS inhibitor (L-NAME - Nω-nitro-L-arginine-methyl-ester) also induced increase of pressure^14^.

The eNOS is a key enzyme that regulates the appropriate endothelial function not only by synthesis of main vasodilator - nitric oxide (NO) - but also by maintaining the equilibrium between the level of NO and ROS. During a reaction catalyzed by eNOS, oxygen binds with a heme group of the enzyme, thus forming a complex that may either oxidize L-arginine to L-citruline which is accompanied by NO secretion or may disintegrate, thus forming a superoxide anion (O_2_^∙−^)^15^. The phenomenon of impairment of the proper eNOS function occur in as young as four-week SHR rats. The over-reactivity of eNOS linked with the over-production of ROS is typical for endothelial dysfunction^16^ and may be one of the major causes of hypertension development in this model^13,17^. The pathological over-expression of eNOS with cause production of superoxide anion (O_2_^∙−^) occurs also in other models of hypertension, such as hypertension induced by aorta bending or in spontaneously hypertensive stroke-prone rats (SHRSP). Undoubtedly, the impaired function of eNOS may be one of the key risk factors of hypertension^18,19^.

No significant response to L-NOARG infusion in SHR treated with egg-lecithin while the control SHR (SHR-C) are responding significantly to L-NOARG infusion may correspond to an intermediate manifestation of the early stage of endothelial dysfunction. Considering the above, it may be also hypothesized that the hypotensive effect of the egg-lecithin (SL) might be related to regulation of eNOS activity.

Diminished activity of eNOS linked with the egg-lecithin (SL) may result from its antioxidative effect. Reduced tyrosine nitration of proteins observed in both hypertensive and normotensive rats points to antioxidative effect of the analyzed preparation. A significant 30% decrease of nitrotyrosine level was observed in both strains of rats treated with egg-lecithin. Nitrotyrosine is a product of the reaction of reactive nitrogen species (RNS) that are formed upon oxidative stress and increased concentration of reactive oxygen species (ROS)^20^. It seems that polyunsaturated fatty acids play a significant role in the antioxidative effect. The scientific community is however divided because PUFAs are claimed to participate in lipid per-oxidation in oxidative stress pathology. This is linked with the presence of unsaturated double bonds between carbon atoms (C=C) in lipid chains that – from the chemical point of view – are reactive and easily form with ROS unstable radical lipid forms. This effect is especially crucial for the central nervous system, of which DHA is the main structural component^21^. In turn, other reports indicate the positive effect of omega-3 PUFAs in oxidative stress reduction^22,23^, which might be related to the regulation of antioxidative enzymes activity by PUFAs. Other studies show that lecithin itself has anti-oxidative activity. In hypertensive patients, phosphatidylcholine reduces oxidative stress in cell membranes metabolism^24^. Investigations with rodents prove that fish-derived PUFAs contribute to increased activities of catalase (CAT), superoxide dismutase (SOD) and glutathione peroxidase (GPX)^25–28^.

**Figure 2:**
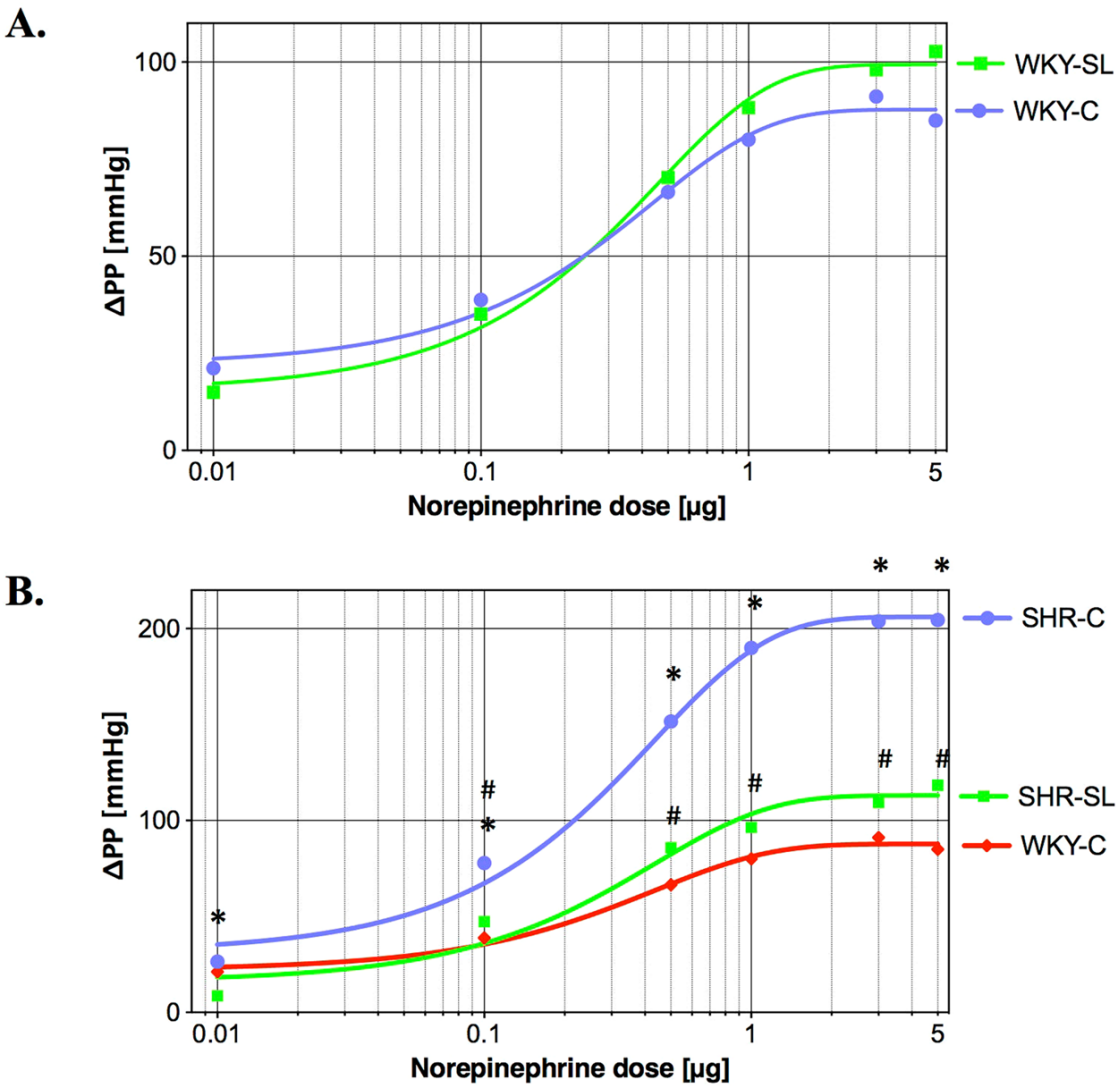
Dose-response curves to norepinephrine. (**A**) Dose-response curves for WKY rats treated with egg-lecithin (WKY-SL; n = 11) and not treated control group (WKY-C; n = 11). (**B**) Dose-response curves for SHR treated with egg-lecithin (SHR-SL; n = 15), SHR not treated control group (SHR-C; n = 15) and WKY not treated control group (WKY-C; n = 11). *P < 0.05 for SHR-C vs. SHR-SL. ^#^P > 0.05, no difference between WKY-C vs. SHR-SL.

The increase of affinity of α-adrenergic receptors of vascular smooth muscle of blood vessels in SHR rats is known to induce excessive norepinephrine vasoconstriction^29,30^. The egg-lecithin (SL) did not influence the responsiveness of mesenteric artery to exogenous norepinephrine in normotensive rats, which was indicated by the lack of significant differences in the response to stimulation of α-adrenergic receptors of the smooth muscle of artery between WKY (Fig. 2A). Opposite observations were made between hypertensive groups of SHR rats where the egg-lecithin significantly attenuate the vasopressor response of mesenteric artery to all doses of norepinephrine (0.01–5.00 μg; Table 3). Additionally, no statistical differences were found in response to norepinephrine beginning from the second dose between the group of SHR rats treated with egg-lecithin (SHR-SL) and the reference normotensive strain (WKY-C). The analysis of dose-response curves indicates a significant lowering of the curve plotted for SHR-SL group towards the approximated curve for WKY-C group (Fig. 2B).

The beneficial effect of the egg-derived lecithin (SL) is manifested in the suppression of the over-reactivity of the mesenteric artery in response to hormonal stimulation in the hypertension. This suppression – resulting from supplementation of egg-lecithin – is so significant in hypertension of SHR that it makes the response induced by norepinephrine statistically the same as in normotensive not treated WKY rats (WKY-C).

The inhibition of vasoconstrictive response to norepinephrine, determined by the effect of the egg-lecithin supplementation, may be linked with the presence of omega-3 fatty acids in its composition. Similar results were obtained in studies with mice where diet supplementation with ω-3 PUFAs contributed to a reduced vasoconstriction to exogenous norepinephrine^31^. Similar results were also show in research addressing the effect of PUFAs from fish on arterial hypertension in SHR rats. Diet supplementation with fish oil (for 12 weeks) in SHR rats (aged 16 weeks) reduced arterial pressure and reactivity of the vascular bed of the mesenteric artery in response to norepinephrine^32^. This mechanism may be related to the presence of DHA in the egg-lecithin (SL) which contributes to reduced expression of cyclooxygenase-2 (COX-2)^33^. An interesting observation was made in the *ex vivo* study on isolated hamster aortas, where improved tension and vasodilatation response was determined in the group of healthy animals receiving DHA for six weeks. Authors of this study linked this effect precisely with suppressed expression of COX-2^34^. In contrast, some reports are available that indicate the lack of DHA effect on vasoconstrictive capabilities in response to norepinephrine in SHR rats^35^, which may suggest the involvement of other constituents of the preparation in the observed suppression of α-adrenergic receptors-dependent vasoconstriction.

In addition, the egg-lecithin reduces also artery vasoconstriction of hypertensive rats (SHR) in response to potassium chloride (KCl). Again, there is no difference in the reaction between hypertensive rats fed by the enriched diet (SHR-SL) and the reference normotensive WKY strain (WKY-C). The reaction of artery induced by potassium chloride is an endothelium-independent response. The effect of vasoconstriction induced by KCl injection is due to the depolarization and regulation of the activity of ionic channels^30,36^. Again, the observed dependency may be ascribed to the role of DHA, because it is known that it affects the value of blood pressure by regulating the potassium channels BK complexes activity of the vascular smooth muscles^37,38^, which in turn may explains the improvement of mesenteric artery response to potassium chloride injection in hypertensive rats as a result of SL treatment.

The analyzed phospholipid fraction (SL) derived from new-generation of eggs exhibits also anti-inflammatory effect by reduction of adhesive molecules (ICAM-1; E-selectin) and inflammatory cytokines (TNF-α; MCP-1) in serum level of SHR. The inflammatory condition plays a highly significant role in the regulation of endothelial function and hypertension, by inducing enhancement of pathological processes and complications. It is known that ω-3 PUFAs affect the reduction of E-selectin, ICAM-1 and VCAM-1 expression by endothelial cells^39,40^. In turn, ICAM-1 and E-selectin participate in the regulation of inflammatory processes and may affect the pathology of endothelial dysfunction by activation of neutrophils and monocytes^41,42^; their activity is also related to increased concentration of pro-inflammatory cytokines like TNF-α and MCP-1. The receptor stimulation of pro-inflammatory cytokines and adhesive molecules is a mechanism of the immune response that initiates a cascade of events augmenting the inflammation in pathology of hypertension. The inflammatory process itself is highly complex, however a transcription protein complex NF-κB is a common element of the analyzed pro-inflammatory factors. An overview of literature data indicates that reduced expression of the inflammatory factors related to PUFAs effect, including DHA, is due to inhibition of NF-κB activity^40,43^. It is, therefore, justified to hypothesize that the anti-inflammatory effect of the egg-lecithin could depend on NF-κB inhibition which has a direct impact on ICAM-1, TNF-α and MCP-1 expression^44–47^. Antioxidative effect of the egg-lecithin may be related of inflammatory markers reduction, as the reactive oxygen species concurrent with hypertension facilitate the expression of such pro-inflammatory factors as: E-selectin, ICAM-1, VCAM-1, MCP-1 and TNF-α, by endothelium^48^. Furthermore, the anti-inflammatory effect of egg-lecithin may again be linked with a low ratio of omega-6 to omega-3 fatty acids, because the low ratio is known to suppress the inflammation^12^.

Undoubtedly, the reduction of the inflammation is directly related to the pathology of arterial hypertension in SHR rats. A decrease in arterial blood pressure is observed along with decreasing concentration of MCP-1 and E-selectin, which has been confirmed by literature data^49^ and by correlations presented in this work. In the hypertensive rats, the inflammation affects also the heart rate, as the statistical analysis in our study demonstrated a positive correlation between levels of TNF-α. MCP-1 and E-selectin and the heart rate. Moreover, the observed correlations confirm the significance of TNF-α. MCP-1 and E-selectin in vasoconstrictor ability of the arterial smooth muscles dependent on α-adrenergic receptors. For each of the indicated inflammatory markers a positive correlation was demonstrated with the vasoconstriction in response to four of the six doses of exogenous norepinephrine.

The effect of egg consumption on cardiovascular diseases has been studied for years. Literature studies show that egg consumption by healthy subject could improve lipid profile and decrease diastolic blood pleasure^50^. This effect could be related to the egg white though. Since it is shown that egg protein decreases blood pressure throughout inhibition of angiotensin-converting enzyme (ACE)^51^. The egg peptides – ovotransferrins – reduce endothelial cell inflammation and endothelial dysfunction. In vascular smooth muscle cells, the egg derived proteins through NF-κB pathway attenuate inflammation^52^. Other studies demonstrate that the consumption of eggs have no influence on the blood pressure and plasma lipids^53^. Studies with the SHR show that addition of egg phospholipids (emulsion of egg yolk) enhance the hypotensive activity of ovokinin (peptide derived from egg white)^54^. Clinical trials with metabolic syndrome patients demonstrate that supplementation by egg derived phospholipids improve endothelial function and reduce daytime systolic blood pressure^55^. Thus, as a potential future research perspective, it may to be interesting to carry out the additional research with phospholipids derived from regular egg.

This work shows that the lecithin (SL) derived from new-generation hen eggs exhibits hypotensive effect by reducing mean values of systolic and diastolic blood pressure in hypertensive SHR rats by 20.95 mmHg and 17.36 mmHg, respectively. Moreover, egg-lecithin in pathology of hypertension of SHR rats demonstrate anti-inflammatory effect and reduce arterial vasoconstriction in response to exogenous norepinephrine. In addition, the egg-derived lecithin was proved to reduce heart rate and decrease the oxidative stress in both normotensive and hypertensive rats’ strains. Furthermore, all effect of the egg-lecithin (SL) are unquestionably deemed as positive in the prevention and therapy of cardiovascular diseases. Components of the investigated phospholipid complex demonstrate various biological activity. The PUFAs (ω-3) of various origins are known to reduce arterial blood pressure and have beneficial effect upon coronary blood vessels in SHR rats^32,35^. Diet supplementation with ω-3 fatty acids increases synthesis of vasodilator prostaglandins as well as decreases activity of angiotensin-converting enzyme and pro-inflammatory cytokines^56^. Undoubtedly, functionality of the egg-derived lecithin (SL) is determined by its complexity. Each of its components contributes to observed beneficial effect of the egg-lecithin (SL). Our egg-lecithin (SL) has a large potential and may be applied in prophylaxis of arterial hypertension and it may also complete standard therapy of hypertension. Nevertheless, this study was conducted on experimental animal models and it requires clinical trials.

## Methods

### Egg derived lecithin

The egg phospholipids, the so-called egg-lecithin or super lecithin (SL), is a mixture of phosphatidylcholine and phosphatidylethanolamine esterified with omega-6 (ω-6) and omega-3 (ω-3) family fatty acids, including: α-linolenic acid (ALA), eicosapentaenoic acid (EPA) and docosahexaenoic acid (DHA). The content of omega-6 fatty acids in this fraction was 15.79% and of omega-3 fatty acids was 10.82% (DHA constitutes 7.12%). The main component of the analyzed phospholipid fraction (81.72% of the whole fraction) was phosphatidylcholine (Supplementary Table S1). Yolks of new-generation hen eggs produced by enrichment of standard feed mixture for Lohmann Brown laying hens in 3% of fish oil, 3% of linseed oil, 1.5% of dried algae, 2% of Humokarbowit preparation^57^, 2% of Humobentofet preparation^58^ and 0.01% of vitamin E, were the sources of phospholipids. Egg yolks were subject to drying process, then phospholipids (egg-lecithin) were extracted with organic solvents of liquid carbon dioxide (supercritical extraction)^59,60^.

Fraction composition was analyzed with the technique of gas chromatography/mass spectrometry (GC/MS). Fraction components were derivatized to volatile methyl esters of fatty acids in the medium of a methanolic solution of 0.5 M NaOH, with a 14% methanolic solution of BF_3_ used as a catalyst. The resultant derivatives of fatty acids were extracted from the reaction mixture with hexane that was finally evaporated under reduced pressure. Afterwards, the methyl esters were dissolved again and analyzed using a GC6890 gas chromatograph coupled with a 5983 MS mass spectrometer (Agilent Technologies Inc. CA, USA) equipped in a quadrupole mass detector. The mixture was separated in an HP-88 capillary column (0.25 mm × 100 m) filled with a cyanopropyl-aryl polysiloxane bed (88:12) with grain size of 0.2 µm. Helium was used as the mobile phase (flow rate 1 ml/min), samples were injected in the split mode (split 4:1). Temperature programme was as follows: initial temperature 60 °C kept for 2 min, heating at 20 °C/min to 180 °C, 3 °C/min to 220 °C and keeping this temperature for 15 min, and finally heating at the rate of 5 °C/min to 250 °C and keeping this temperature for 8 min; the total time of analysis was 50.33 min. Spectra were identified using the algorithm of searching the National Institute of Standards and Technology (NIST) library (version of 2008).

### Animals and treatment

The experiment was conducted with 30 males of spontaneously hypertensive rats (SHR/NCrl; Charles River Laboratories, Hamburg, Germany) and 22 males of Wistar Kyoto rats (WKY/NCrl; Charles River Laboratories, Hamburg, Germany). Experimental protocol was approved by the I Local Ethical Commission at the Institute of Immunology and Experimental Therapy of the Polish Academy of Sciences in Wroclaw (approval no. 61/2010) and was performed in accordance with the relevant guidelines and regulations. Chow used in the experiment was poor in polyunsaturated fatty acids (PUFA pure; Labofeed B without linseed fraction, Kcynia, Poland; composition in Supplementary Table S2), to exclude the potential effect of PUFAs from other sources on the results of experiment. Egg-lecithin (SL) was administered with the chow. The enriched chow contained 6% of egg phospholipids, by this mean that experimental chow contained: 4.90% of phosphatidylcholine, 0.65% of omega-3 and 0.95% of omega-6 fatty acids.

At the age of 7 weeks, the rats were divided into 4 groups: SHR control group (SHR-C) receiving PUFA pure chow (n_SHR-C_ = 15); SHR experimental group (SHR-SL) receiving SL with PUFA pure chow (n_SHR-SL_ = 15); WKY control group (WKY-C) receiving PUFA pure chow (n_WKY-C_ = 11); WKY experimental group (WKY-SL) receiving SL with PUFA pure chow (n_WKY-SL_ = 11). The access to chow and water was provided *ad libitum*. The amount of eaten chow was controlled every two-three day. Calculated mean daily intake of the egg-lecithin was 1.5 g/animal. The animals were kept under constant conditions: temperature of 20 **°**C, humidity of 55 ± 10% and under the 12-h day/night cycle. The rats were treated for 12 weeks. The mean body weight of SHR and WKY rats at the beginning of the experiment was as follows: 198.0 ± 5.8 g and 221.3 ± 8.5 g.

The egg-lecithin (SL) was administered for 12 weeks. Rat body weight was controlled once a week during entire experimental period. Postmortem examination of the rats was performed following the last week of the experiment. Under anesthesia, the blood samples were collected and mesenteric artery was isolated. The animals were then sacrificed by cervical dislocation.

### Measurement of blood pressure and heart rate

Blood pressure and heart rate were measured using rat cardiovascular telemetry system DSI (Data Sciences International Inc., MN, USA). This method is fully automated and used for 24-h pressure registration including day and night activity. The system registers pressure, heart rate and activity. The measurements were taken by implanted transmitter (TA11PA-C40, DSI, MN, USA), which was implanted directly to the abdominal aorta. The data were transferred wirelessly (AM wave) from a transmitter to a receiver (*Rat Pressure & Activity Receiver RPC-1*, DSI, MN, USA), located under the cage and then measurements from receiver were then transferred to a computer through Data Exchange Matrix (DSI, MN, USA).

Regarding to our experience, implanted transmitter for more than 4 weeks could affect the catheter condition of transmitter. Thus, the surgery of transmitter implantation was performed at the 8th week of diet supplementation. Rats were anaesthetized by izoflurane inhalation. After 7-day of recovery period, a series of measurements were carried out. Blood pressure was measured between the 9^th^ and the 12^th^ week of the experiment, including four to five 24-h measuring cycles (a day and night cycle) performed in each animal. The time intervals between measuring cycles were three to four days.

#### Reactivity of mesenteric artery in perfusion *ex vivo*

Rats were anaesthetized with a ketamine at dose of 0.1 mg/g BW (Bioketan, Biovet Vetoquinol, Gorzów Wielkopolski, Poland). The mesenteric artery was dissected according to the method described by D.D. McGregor^61^. The isolated mesenteric artery with the vascular bed was placed in a perfusion apparatus (UP-1000, type 834 by Harvard Apparatus, USA). The peristaltic pump with constant speed of 8.4 ml/min perfused *ex vivo* the mesenteric vessel with Krebs solution, having the following composition (mM): NaCl 112.0, KCl 5.0, NaH_2_PO_4_ 1.0, MgCl_2_ 0.5, CaCl_2_ 2.5, NaHCO_3_ 25.0, D( + ) glucose 11.2 ^62^. The solution was saturated with oxygen (95% O_2_, 5% CO_2_) and heated to 30 °C. Its osmolarity reached 284 mOsm, and its pH was 7.4. Perfusion pressure (PP) was registered by APT 300 pressure transducer and monitored by TAM-D type 705/2 (Hugo Sachs Electronic, March-Hugstatten, Germany).

#### Stage 1

Once the perfusion pressure (PP) had stabilized, a series of injections was performed with increasing doses of norepinephrine (NE): 0.01 μg, 0.1 μg, 0.5 μg, 1.0 μg, 3.0 μg, and 5.0 μg. NE administered in injections (expressed in μg) was dissolved in 100 µl of Krebs solution. Changes in the resistance of the vascular bed (changes in PP - ΔPP) were registered for each dose of the drug.

#### Stage 2

In the successive stage of the experiment, NE dissolved in Krebs fluid was administered in continuous infusion in the concentration of 0.5 μg/ml (*NE infusion*), which caused an increase of the basal perfusion pressure – PP.

#### Stage 3

After 60 min, the inhibitor of nitric oxide synthase (L-NOARG, N-ω-nitro-L-arginine) was added to the perfusion solution in a dose of 1.0 mg/ml (*NE* + *L-NOARG infusion*). In the last stage of the experiment, potassium chloride (KCl) was injected in the concentration of 10.5 mg/ml.

Perfusion pressure (PP [mmHg]) was monitored at all stages of perfusion analysis. All solutions were prepared *ex tempore*. The following pharmaceuticals were used in the perfusion analysis: norepinephrine (Levonor, Polfa S.A. Warsaw, Poland); acetylcholine (Fluka, Seelze, Germany); L-NOARG (Sigma Chemical Co. St. Louis, MO, USA), and potassium chloride (Polfa S.A. Warsaw, Poland).

### Biochemical analyses in blood serum

The blood serum levels of soluble intercellular adhesion molecule-1 (sICAM-1), soluble E-selectin (sE-slelectin), tumor necrosis factor-α (TNF-α), monocyte chemoattractant protein-1 (MCP-1) and nitrotyrosine, were analyzed by commercial kits. The level of nitrotyrosine was determined with a Nitrotyrosine ELISA kit (no. 17–376, Merck, MA, USA). The contents of cytokines were assayed using a multi-parametric fluorescence-laser system (Luminex). In turn, sE-selectin and sICAM-1 were assayed using the Milliplex Rat Cardiovascular Disease (CVD) Panel kit (Millipore, Billerica, MA, USA). Levels of MCP-1, TNF-α, were assayed using the Milliplex Rat Expanded Cytokin Magnetic kit (Millipore, Billerica, MA, USA). All tests were performed according to the instructions provided by kits. Data was read out and analyzed with the FlexMap 3D, Luminex xPONENTH 4.0 Software and Luminex Analyst Program (Luminex Corporation, TX, USA).

### Statistical analysis

Data is express as arithmetic means ± standard error of the mean (SEM). The comparisons between groups were made using the Student’s t-test or Man-Whitney U-test. While, changes within the groups (depended variables) was analyzed using depended Student’s t-test or Wilcoxon test. Relationships between the analyzed parameters were evaluated with Pearson’s linear correlation or Spearman rank correlation. The selection of the appropriate statistical test was determined by meeting assumptions for parametric test. P values < 0.05 were considered as statistically significant. The statistical analysis was conducted with STATISTICA 10 software (StatSoft). Dose-response curves were approximated according to the following equation: y = ΔPP_min_ + (ΔPP_max_ − ΔPP_min_)/(1 + 10^(lgLD50−x)^), where: ΔPP_max_ – maximal approximated value on the Y-axis; ΔPP_min_ – minimal approximated value on the Y-axis; EC50 – concentration of the pharmaceutical ensuring half the response between ΔPP_max_ and ΔPP_min_ values. Parameters of approximated dose-response curves were calculated in GraphPad Prism 6 software.

### Data Availability

The datasets generated during the current study are available from the corresponding author on reasonable request.

## Electronic supplementary material

Supplementary Table S1 and Table S2
