# Supplementary material for: Lecithin derived from ω-3 PUFA fortified eggs decreases blood pressure in spontaneously hypertensive rats
Source: Sci Rep. 2017 Sep 28;7:12373. doi: 10.1038/s41598-017-12019-w (PMC5620069; doi:10.1038/s41598-017-12019-w)
Supplement: Supplementary file 1 — Supplementary Table S1 and Table S2 [file 41598_2017_12019_MOESM1_ESM.pdf]

Supplementary Information

Lecithin derived from ω-3 PUFA fortified eggs decreases blood pressure in spontaneously hypertensive rats

Nowacki D., Martynowicz H., Skoczyńska A., Wojakowska A., Turczyn B., Bobak Ł., Trziszka T., Szuba A.

Supplementary Table S1. The percentage of the fatty acids in the phospholipid fraction isolated from egg yolk.

**Phospholipid fraction**		**[%]**
Purity as a substance insoluble in acetone		73.00
Phosphatidylcholine		81.72
Phosphatidylethanolamine (PE)		18.27
**The fatty acid profile of the phospholipid fraction**		**[%]**
Tetradecanoic acid	C14:0	0.40
Tetradecenoic acid	C14:1	0.16
Hexadecanoic acid	C16:0	26.36
Hexadecenoic acid	C16:1	2.52
Heptadecanoic acid	C17:0	0.24
Octadecanoic acid	C18:0	14.05
Octadecenoic acid (kwas oleic; OA)	C18:1	29.66
Octadecadienoic acid (kwas linoleic; LA)	C18:2	13.08
α-linolenic acid (octadecatrienoic; ALA)	C18:3	3.12
Eicosadienoic acid	C20:2	0.18
Eicosatrienoic acid	C20:3	0.12
Eicosatetraenoic acid (arachidonic acid; AA)	C20:4	2.41
Eicosapentaenoic acid (EPA)	C20:5	0.58
Docosahexaenoic acid (DHA)	C22:6	7.12
ω-3		10.82
ω-6		15.79
ω6/ω3		1.46
Saturated fatty acids		41.05
Unsaturated fatty acids		58.95
Polyunsaturated fatty acids (PUFA)		26.61
Monounsaturated fatty acids (MUFA)		32.34

Supplementary Table S2. Nutrition information table of PUFA pure chow (Labofeet B without linseed fraction) and Labofeet B standard chow.

	**Labofeet B without linseed fraction**	**Labofeet B standard**
	**amount per 100 g**	
Energy	272.28 kcal	274.67 kcal
Protein	17.5 g	17.7 g
Carbohydrate	73.81 g	72.79 g
Fiber	6.34 g	6.42g
Total fat	3.14 g	3.87 g
Linseed fat	0 mg	730 mg
PUFA	9.78 mg	506.3 mg
MUFA	< 2.1 mg	129.9 mg
